# Therapeutic effect of recombinant *Echinococcus granulosus* antigen B subunit 2 protein on sepsis in a mouse model

**DOI:** 10.1186/s13071-024-06540-x

**Published:** 2024-11-15

**Authors:** Ya-Yun Qian, Fei-Fei Huang, Si-Yu Chen, Wei-Xiao Zhang, Yin Wang, Peng-Fei Du, Gen Li, Wen-Bo Ding, Lei Qian, Bin Zhan, Liang Chu, Dong-Hui Jiang, Xiao-Di Yang, Rui Zhou

**Affiliations:** 1 First Affiliated Hospital of Bengbu Medical University, Bengbu, 233000 China; 2 Anhui Key Laboratory of Infection and Immunity of Bengbu Medical University, Bengbu, 233000 China; 3 First People’s Hospital of Changzhou, Changzhou, 213000 China; 4 Wuxi School of Medicine, Jiangnan University, Wuxi, 214028 China; 5 Department of Critical Care Medicine, Affiliated Hospital of Jiangnan University, Wuxi, 214122 China; 6 National School of Tropical Medicine, Baylor College of Medicine, Houston, TX 77030 USA; 7 Second Affiliated Hospital of Bengbu Medical University, Bengbu, 233000 China; 8 Department of Critical Care Medicine, First People’s Hospital of Haidong, Haidong, 810600 China

**Keywords:** Sepsis, *Echinococcus granulosus*, Antigen B (*Eg*AgB), Immune response, Macrophage

## Abstract

**Background:**

Sepsis is a potentially fatal systemic inflammatory response syndrome (SIRS) that threatens millions of lives worldwide. *Echinococcus granulosus* antigen B (*Eg*AgB) is a protein released by the larvae of the tapeworm. This protein has been shown to play an important role in modulating host immune response. In this study we expressed *Eg*AgB as soluble recombinant protein in *E. coli* (r*Eg*AgB) and explored its protective effect on sepsis.

**Methods:**

The sepsis model was established by cecal ligation and puncture (CLP) procedure in BALB/c mice. The therapeutic effect of r*Eg*AgB on sepsis was performed by interperitoneally injecting 5 µg r*Eg*AgB in mice with CLP-induced sepsis and observing the 72 h survival rate after onset of sepsis. The proinflammatory cytokines [tumor necrosis factor (TNF)-α, interleukin (IL)-6] and regulatory cytokines [IL-10, transforming growth factor beta (TGF-β)] were measured in sera, and the histopathological change was observed in livers, kidneys, and lungs of septic mice treated with r*Eg*AgB compared with untreated mice. The effect of r*Eg*AgB on the macrophage polarization was performed in vitro by incubating r*Eg*AgB with peritoneal macrophages. The levels of TLR2 and MyD88 were measured in these tissues to determine the involvement of TLR-2/MyD88 in the sepsis-induced inflammatory signaling pathway.

**Results:**

In vivo, we observed that treatment with r*Eg*AgB significantly increased the survival rate of mice with CLP-induced sepsis up to 72 h while all mice without treatment died within the same period. The increased survival was associated with reduced pathological damage in key organs such as liver, lung, and kidneys. It was supported by the reduced proinflammatory cytokine levels and increased regulatory cytokine expression in peripheral blood and key organ tissues. Further study identified that treatment with r*Eg*AgB promoted macrophage polarization from classically activated macrophage (M1) to regulatory M2-like macrophage via inhibiting TLR2/MyD88 signal pathway.

**Conclusions:**

The therapeutic effects of r*Eg*AgB on mice with sepsis was observed in a mice model that was associated with reduced inflammatory responses and increased regulatory responses, possibly through inducing polarization of macrophages from proinflammatory M1 to regulatory M2 phenotype through inhibiting TLR2/MyD88 inflammatory pathway.

**Graphical Abstract:**

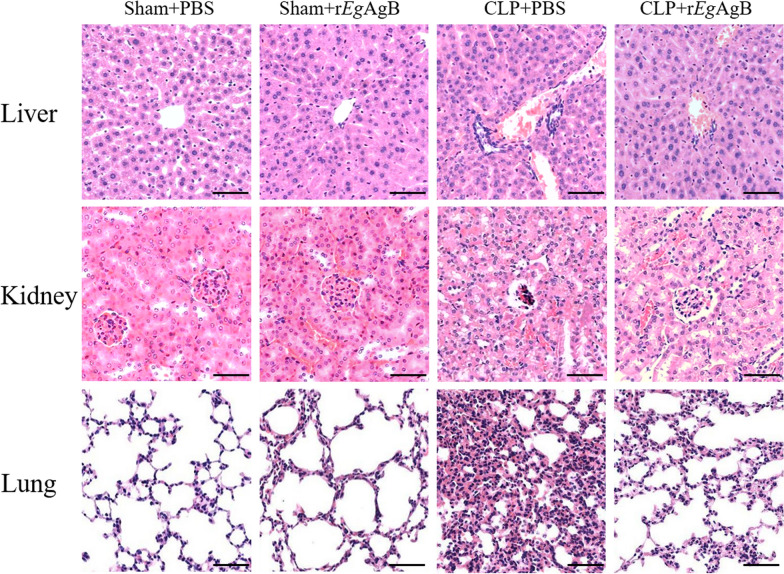

**Supplementary Information:**

The online version contains supplementary material available at 10.1186/s13071-024-06540-x.

## Background

Sepsis is a life-threatening complication caused by the overwhelming immune response to bacterial infection [1, 2]. It is estimated that there are nearly 50 million cases of sepsis worldwide annually, and the mortality rate of sepsis is as high as 30–70% [3, 4]. High mortality and high treatment cost place it as a great burden on the healthcare and world economy.

Sepsis involves multiple organ injuries, most commonly in liver, kidney, and lung, which can lead to acute damage and functional failure of these key organs and eventual dead [5–7]. The pathophysiological mechanism of sepsis is extremely complex and involves many different aspects of inflammatory responses, immune disorders, mitochondrial disorders, coagulation disorders, neuroendocrine abnormalities, and so on, among them, the overwhelming inflammatory responses including the proinflammatory cytokine storm are the cornerstone of sepsis and the major cause of death [8]. A series of repeated stimuli from pathogens promote host macrophages to phagocytose pathogens and produce a lot of proinflammatory cytokines, mostly the tumor necrosis factor (TNF)-α and interleukin (IL)-6, which play an important role in the development of systemic inflammatory response [9, 10]. This acute immune response is followed by the release of IL-10, transforming growth factor beta (TGF-β), and other antiinflammatory cytokines as a protective mechanism against inflammation-caused pathology and damage especially in key organs [11]. If this strong inflammatory response gets out of control, it will lead to organ dysfunction and death. Therefore, how to control the overwhelming release of proinflammatory cytokines and balance the immune responses has been considered as an important strategy to treat sepsis and reduce its mortality.

Macrophages are a class of innate immune cells derived from monocytes, which are distributed in different tissues throughout the body and regulate homeostasis and adaptive immune responses [12]. Macrophages activated by interferon gamma (IFN-γ) or lipopolysaccharide (LPS) are known as M1 macrophages, while those activated by IL-4 and IL-13 are called alternatively activated macrophages (AAM) or M2 cells [13]. Under conditions of sepsis, macrophages respond rapidly to the infection and are activated to M1, kill and eliminate pathogens through phagocytosis and secretion of inflammatory cytokines, constituting the body’s first line of defense against exogenous infection [14, 15]. M1 macrophages highly express proinflammatory cytokines, including TNF-α, IL-1β, IL-6, inducible nitric oxide synthase (iNOS), and reactive oxygen species (ROS), promoting inflammatory responses to clean invaded pathogens, at the same time aggravating tissue immunological damage and organ dysfunction [16]. Some M2 macrophages are not activated by IL-4 and IL-13, but express high levels of IL-10, TGF-β, and arginase-I (Arg-I) involved in the regulation and control of immune response and the tissue repair and tissue remodeling [17, 18] This type of M2 macrophage is called M2-like macrophages [19].

In normal immune response, M1 and M2 phenotype macrophages are well orchestrated and highly regulated [20]. Induction of M2-like macrophages has been found to attenuate hyper-inflammation and immunopathology in sepsis [21, 22]. Therefore, regulation and balance of macrophage M1 and M2 during sepsis may have important implications for controlling septic infection and reducing mortality.

Toll-like receptors (TLRs) are a family of receptors expressed on the surface of immune cells and involved in cell recognition [23]. TLRs play crucial roles in pathogen recognition and host immune response and are closely related to the occurrence and development of sepsis [24, 25]. As an important part of the TLRs family, TLR2 is mainly expressed on lymphocytes and macrophages. It has remarkable characteristics in recognizing pathogens and initiating immune responses and inflammation. When TLR2 binds to pathogen-associated molecular patterns (PAMP), it activates downstream transcription factors such as NF-κB through the linker molecule MyD88 to initiate the expression of inflammatory mediators, thereby promoting the immune system to initiate cellular and humoral immunity against pathogens. The signaling of TLR2/MyD88 pathway is strictly controlled. If this pathway is continuously activated, it will lead to excessive release of inflammatory mediators and trigger severe inflammatory response and even autoimmune diseases, causing tissue damage. Blocking the expression and activation of TLR2 can inhibit the release of inflammatory factors and avoid the occurrence of excessive inflammatory responses [26].

It has been identified that helminth infections not only cause disease but also participate in regulating or modulating host immune response as a survival strategy. Helminth secretes a variety of proteins that interact with host immune system and play immunomodulatory role in reducing host hypersensitivity status. As a result, helminth infection or helminth-derived proteins have been experimentally used in the treatment of inflammatory or autoimmune diseases [27–30]. Further studies demonstrated that helminth-derived proteins stimulated regulatory T cells (Tregs) and the release of antiinflammatory factors such as TGF-β and IL-10 to reduce excessive inflammatory responses [31]. Helminth infection or its derived proteins were also able to modulate macrophages to M2-like macrophages as an approach to treat inflammatory diseases including sepsis [32].

Echinococcosis is a serious parasitic disease caused by tapeworms *Echinococcus spp*. It includes two main types of the disease: cystic echinococcosis and alveolar echinococcosis [33]. Cystic echinococcosis (CE) is a severe zoonotic parasitic disease caused by the larval stage of *Echinococcus granulosus*. When humans are infected with *E. granulosus*, it forms many encysted cysts in the body, most in the liver and lungs [34]. The hydatid cyst contains a variety of proteins secreted by the parasitic larvae, and antigen B (*Eg*AgB) is the main component of the proteins secreted in the cyst fluid. It is a polymeric lipoprotein with 160 kDa in molecular size, which can be disassociated to several subunits with molecular weights of 8, 16, 24, and 32 kDa. Total five subunits have been identified that are encoded by five gene subfamilies (*Eg*AgB8/1-*Eg*AgB8/5) with genetically related 8-kDa subunit monomers. Even though these subunits are genetically related, each of them contains sequence variation and different structure [35]. *Eg*AgB is highly specific and immunogenic during infection, in which *Eg*AgB subunit 2 is more immunogenic and specific than others, and is therefore used as immunodiagnostic antigens [35–37]. Except for its immunodiagnostic property, a recent study revealed that *Eg*AgB was able to bind to monocytes and macrophages to reduce their inflammatory responses, indicating its immunomodulatory function on host immune system [37]. The results are consistent with our previous study that demonstrated the potential role of *E. granulosus* cyst fluid (*Eg*CF) in promoting M2-like macrophage polarization and modulating the inflammatory response [38]. Further study using *Eg*AgB demonstrated its therapeutic effect on inflammatory bowel disease by regulating intestinal fora or microbiome and modulating macrophage differentiation toward M2-like [39]. In this study we would like to further explore the therapeutic potential of *Eg*AgB, using recombinant *Eg*AgB subunit 2 protein as a representative in sepsis based on its potential immunomodulatory property.

## Methods

### Animals

The specific pathogen-free male BALB/c mice (6–8 weeks old with weight of 18–22 g) were purchased from the Animal Center of Bengbu Medical College. All mice were maintained in a facility with temperature controlled between 20 °C and 25 °C and free access to food and water. All animals were handled and raised in accordance with the Ethics Committee guidelines of the Bengbu Medical College.

### Expression and purification of recombinant *Eg*AgB protein (r*Eg*AgB)

The DNA encoding *Eg*AgB (GenBank: ACZ51457) was amplified from the total cDNA of *Echinococcus granulosus* hydatid cysts, and cloned in frame into *E. coli* expression vector pPET-28a( +) using EcoRI and XhoI restriction sites [40]. The constructed plasmid *Eg*AgB/pPET-28a( +) was transformed into expression host *E. coli* BL21(DE3). The r*Eg*AgB with a His tag at C-terminus was expressed under induction of 1 mM IPTG and purified by immobilized metal ion affinity chromatography (IMAC) using a nickel column. The contaminated endotoxin was removed using an endotoxin removal kit (ToxOut Endotoxin Removal Kit) (BioVision, USA) and the residual endotoxin was measued by ToxinSensor Chromogenic LAL Endotoxin Assay Kit (GenScript Biotech, Nanjing, China). The purified r*Eg*AgB was stored in PBS buffer without imidazole and quantitated using BCA protein quantification kit (Biosharp, Hefei, China) and analyzed by SDS-PAGE before being stored at −80 °C until use.

### Isolation and culture of mouse peritoneal macrophages

Peritoneal macrophages (PMs) are one of the most studied macrophage populations and play an important role in the control of pathological processes such as infection and inflammation [41]. To extract PMs from healthy mice, BALB/c mice were sacrificed and 5 mL pre-cooled RPMI 1640 medium was injected into peritoneal cavity of each mouse. The peritoneal lavage solution was extracted. The cells were pelleted by centrifugation at 500 × g, 4 ℃ for 5 min and resuspended in complete 1640 medium containing 10% fetus bovine serum. Peritoneal macrophages were incubated in RPMI at 37 °C for 3 h, non-adherent cells were removed and PMs were obtained by their adhesion properties [42].

### Effect of r*Eg*AgB on macrophage polarization in vitro

PM cells were collected from the adherent peritoneal cells after being cultured in RPMI 1640 for 3 h. Collected PMs were divided into four groups with 1 × 10^6^ cells for each group: (i) control group incubated with complete 1640 medium (RPMI 1640); (ii) incubated with r*Eg*AgB (1 μg/ml) (r*Eg*AgB); (iii) incubated with LPS (100 ng/ml) (LPS); and (iv) incubated with LPS (100 ng/ml) in the presence of r*Eg*AgB (1 μg/ml) (r*Eg*AgB + LPS). Cell culture supernatant and cells were collected 24 h after incubation. The levels of TNF-α, IL-6, IL-10, and TGF-β in the culture supernatant were detected using individual enzyme linked immunosorbent assay (ELISA) detection kits (Mouse TNF-α, IL-6, and IL-10 ELISA Kits from Dakewe Biotech, China and Mouse TGF-β ELISA Kit from ABclonal, USA). The M1-related marker CD86 and M2-related marker CD206 on the cultured PMs were measured by flow cytometry.

### Flow cytometry

The cultured PMs were treated with fixable viability dye efluor 510 (BioLegend, USA) for 10 min to differentiate dead/live cells, then blocked with Fc receptor blocker for 10 min. Cells were then incubated with FITC-anti-F4/80, BV605-anti-CD11b, and APC-anti-CD86 antibodies (BioLegend, USA) for 25 min for surface marker staining. After being fixed and permeabilized by using Thermo Fixation/Permeabilization Kit (Thermo Fisher Scientific, USA), the cells were stained with PE-anti-CD206 (BioLegend, USA) for 30 min. All labeling steps were performed in the dark. The isotype-matched immunoglobulins (BioLegend, USA) and FMO were used as a control for nonspecific staining as a baseline. Flow cytometry acquisition was performed with DxP Athena™ flow cytometer (Cytek Biosciences Inc., CA, USA) and data were analyzed using FlowJoRV10 software.

### Establishment of mouse sepsis model by cecal ligation and puncture (CLP)

To induce the clinical course of sepsis, CLP surgery was performed in mice to establish mouse sepsis model [43]. Male BALB/c mice were fasted for 12 h with only access to water. After being anesthetized with isoflurane inhalation, the mice were stabilized in the supine position and the abdominal cavity was opened. The cecum was pulled out and ligated at about 1.0 cm from the end of the cecum with 3.0 silk thread, then pierced with an 18-gauge sterile needle to squeeze out a little intestinal content to ensure the exposed infection. The cecum was returned to the abdominal cavity and each layer of opened tissue was sutured with 4.0 silk. The sham control group underwent the same open surgery without CLP. The general condition and survival rate of mice were observed up to 72 h after CLP.

### Treatment of mouse sepsis with r*Eg*AgB

Mice were randomly divided into 4 groups with 16 mice each: (i) sham procedure control group treated with PBS only (sham + PBS); (ii) sham control group treated with r*Eg*AgB (sham + r*Eg*AgB); (iii) CLP group treated with PBS (CLP + PBS); and (iv) CLP group treated with r*Eg*AgB (CLP + r*Eg*AgB). In r*Eg*AgB treatment groups, the mice were intraperitoneally injected with 5 μg of r*Eg*AgB in a total volume of 100 µl 30 min after surgery. In PBS control groups, mice were given with the same volume of PBS after surgery; 12 h after treatment, six mice from each group were euthanized; the serum samples were collected for detecting the levels of cytokines and biological function markers; and the liver, kidney, and lung organs were collected for evaluating the histopathological changes. The remaining ten mice were kept for observation for up to 72 h to calculate the survival rate using the Kaplan–Meier method.

### Serological assays

The levels of proinflammatory cytokines (TNF-α, IL-6) and regulatory cytokines (IL-10 and TGF-β) were measured in sera collected from six mice euthanized 12 h after CLP surgery using the same ELISA detection kits mentioned above. The levels of alanine transaminase (ALT) and aspartate transaminase (AST), as well as the blood urea nitrogen (BUN) and creatinine (Cr) were measured by an automatic chemistry analyzer (Beckman Coulter, Brea, USA) to evaluate sepsis-caused acute injury of liver and kidney, respectively.

### Histopathological examination in tissues of liver, kidney, and lung

Parts of liver, kidney, and lung organs harvested from six euthanized mice in each group was fixed in 4% paraformaldehyde, followed by paraffin embedding, sectioning, and staining with hematoxylin and eosin (HE) for histopathological examination. The pathological changes were observed under the light microscope, six slices were examined for each group, and were scored on the basis of the injury degree in liver listed in Table 1, kidney listed in Table 2 [44], and lung listed in Table 3 [45].

**Table 1:** Liver injury score parameters

Hepatocyte cytoplasmic vacuolation, sinusoidal congestion, hepatocyte necrosis	Injury scores
No pathology	0
< 25% liver involvement	1
25–50% liver involvement	2
50–75% liver involvement	3
˃ 75% liver involvement	4

**Table 2:** Kidney injury score parameters

Tubular injury and glomerular atrophy	Injury scores
No pathology	0
< 25% kidney involvement	1
25–50% kidney involvement	2
50–75% kidney involvement	3
˃ 75% kidney involvement	4

**Table 3:** Lung injury score parameters

Alveolar congestion, alveolar edema, neutrophil infiltration, alveolar septal thickening	Injury scores
No pathology	0
< 25% lung involvement	1
25–50% lung involvement	2
50–75% lung involvement	3
˃ 75% lung involvement	4

### Expression of iNOS and Arg-1 in liver, kidney, and lung tissues

Parts of liver, kidney, and lung tissues harvested from each group were rinsed in physiological saline solution, and then homogenized using an ultrasonic cell crusher. The homogenate of these tissues was obtained by centrifuging at 4500 rpm, 4 °C, for 15 min. The protein concentration in the supernatant was determined using a BCA protein quantification kit, and the levels of iNOS (M1 marker) and Arg-1 (M2 marker) were measured using specific ELISA detection kits (ABclonal, USA).

### Expressions of TLR2 and MyD88 in PMs and tissues of mice

The total proteins were extracted from PMs and tissues of liver, kidney, and lung of mice from each group with RIPA lysis buffer containing 0.1% PMSF and quantitated using BCA Protein Quantitation Kit. Equal amount of protein from each mouse was loaded and separated by 10% SDS-PAGE gels, then transferred to 0.45 um polyvinylidene fluoride (PVDF) membrane. The membranes were blocked with 5% skim milk at room temperature for 3 h and then incubated with rabbit anti-TLR2 antibody (1:1500) (Abcam, Cambridge, UK), or rabbit anti-MyD88 antibody (1:1000) (Affinity Biosciences, Cincinnati, USA) overnight at 4 °C followed by incubation with horseradish peroxidase (HRP)-conjugated secondary antibody secondary antibody (1:5000) (Biosharp, Hefei, China) at room temperature for 1 h. The same amount of tissue lysate was reacted with rabbit anti-β-actin antibody (1:2000) (Cell Signaling Technology, Massachusetts, USA) as baseline control. The specific bands were visualized by chemiluminescent substrate and semi-quantitated by Image Lab System. The results are expressed as ratios of TLR2 and MyD88 to β-actin control.

### Statistical analysis

Data were obtained from at least three independent experiments for all studies. GraphPad Prism version 8.0 software (GraphPad Software, Inc., USA) was used to analyze statistical differences between groups. Results were expressed as the mean ± SEM. Statistical analysis was performed using Shapiro–Wilk normality test, and one-way analysis of variance (ANOVA) followed by Tukey–Kramer or Tamhane’s multiple comparisons test. The difference in survival rates among the groups were compared using Kaplan–Meier survival analysis. *P*-value less than 0.05 was considered as statistically significant.

## Results

### Expression and purification of r*Eg*AgB

r*Eg*AgB was successfully expressed as a soluble recombinant protein in *E. coli* BL21 under induction of 1 mM IPTG and purified with IMAC. SDS-PAGE showed the size of purified r*Eg*AgB as about 10.0 kDa as expected on the basis of the sequence (9.40 kDa) (Supplementary Fig. 1). The level of contaminated endotoxin in IMAC purified r*Eg*AgB (2000 EU/mg) was significantly removed after running through Endotoxin Removal Resin to less than 0.06 EU/mg in the final purified product in PBS.

### r*Eg*AgB reduced LPS-induced macrophage inflammatory responses in vitro

**Fig. 1:**
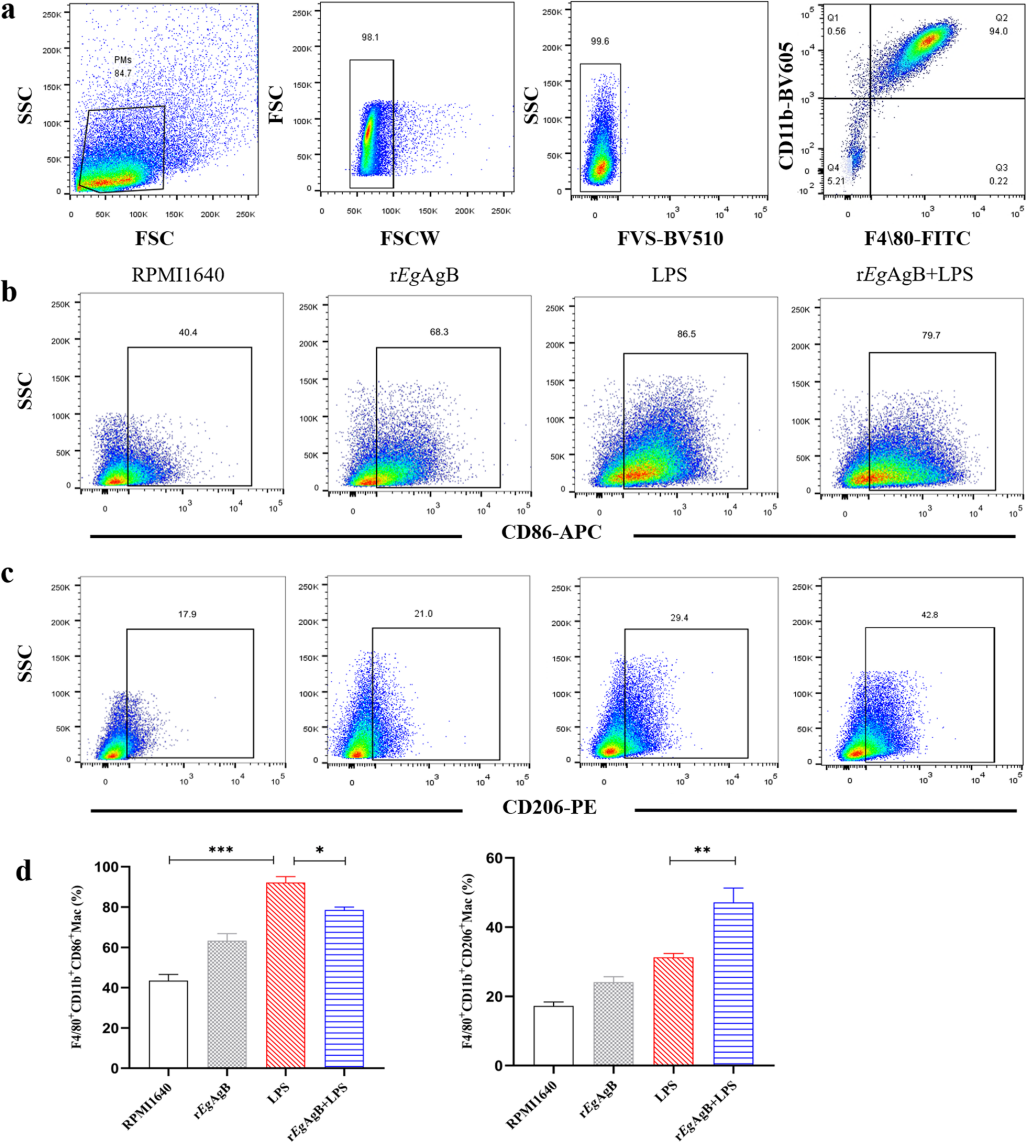
r*Eg*AgB protein inhibited LPS-induced differentiation of M1 macrophages and promoted M2-like type macrophages. **a** The flow cytometry was applied to gate dead cells, adhere cells, and F4/80^+^ peritoneal macrophages (PMs). The PMs were incubated with r*Eg*AgB (1 μg/ml), LPS (100 ng/ml), r*Eg*AgB + LPS, or RPMI1640 medium for 24 h, and the M1-related marker CD86 (**b**, **d**) and M2-related marker CD206 (**c**, **d**) were measured by flow cytometry. The results were shown as the mean ± SEM for each group (*n* = 3). *P < 0.05, **P < 0.01, ***P < 0.001

To observe the effect of r*Eg*AgB on LPS-induced inflammatory responses in PMs, the inflammatory M1-related marker CD86 and the M2-related marker CD206 were measured on these treated PMs using flow cytometry, and flow cytometry with a gating strategy was used to differentiate firstly dead cells and adhered cells and then PMs labeled with CD11b^+^F4/80^+^ (Fig1a). After being induced with LPS, most of peritoneal macrophages expressed F4/80^+^CD11b^+^CD86^+^ (compared with RPMI1640 medium control) (ANOVA: *F* (3, 8) = 54.59, *P* < 0.0001) (Fig. 1b, c, d). Co-incubation with r*Eg*AgB effectively reduced the F4/80^+^CD11b^+^CD86^+^ macrophages stimulated by LPS and increased the F4/80^+^CD11b^+^CD206^+^ macrophages compared with macrophages without r*Eg*AgB treatment (ANOVA: *F* (3, 8) = 54.59, *P* < 0.0001;*F* (3, 8) = 30.66, *P* < 0.0001, respectively) (Fig. 1b, c, d).

**Fig. 2:**
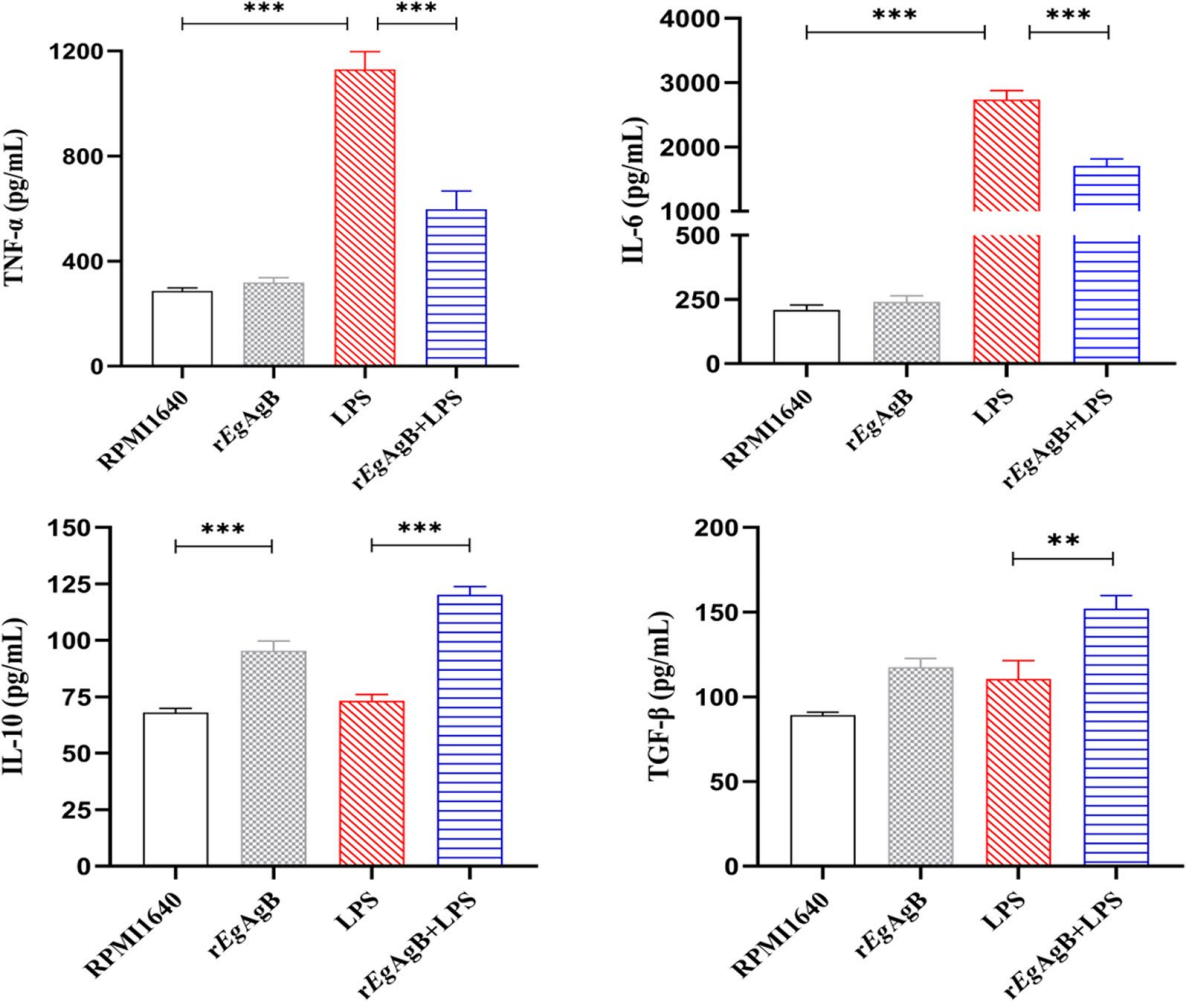
The culture supernatant was collected to measure levels of M1 cytokines (TNF-α, IL-6) and M2 cytokines (IL-10, TGF-β) by ELISA. The results were shown as the mean ± SEM for each group (*n* = 6). *P < 0.05, **P < 0.01, ***P < 0.001

The levels of inflammatory cytokines in the cell culture supernatant of each group were measured by ELISA. LPS-stimulated macrophages secreted higher levels of proinflammatory cytokines (TNF-α and IL-6) compared with RPMI1640 group (Fig. 2). Co-incubation with r*Eg*AgB decreased the levels of proinflammatory cytokines (ANOVA: *F*_(3, 20)_ = 57.20, P < 0.0001; *F*_(3,20)_ = 188.20, *P* < 0.0001, respectively) and enhanced the levels of regulatory cytokines IL-10 and TGF-β (ANOVA:*F*_(3, 20)_ = 53.17, *P* < 0.0001; *F*_(3,20)_ = 15.73, *P* < 0.0001, respectively) (Fig. 2).

All above results indicated that r*Eg*AgB was able to inhibit LPS-induced M1 macrophages and promote their differentiation from M1 to M2-like in vitro*.*

### Treatment with r*Eg*AgB improved the survival rate of CLP-induced *sepsis* in mice

**Fig. 3:**
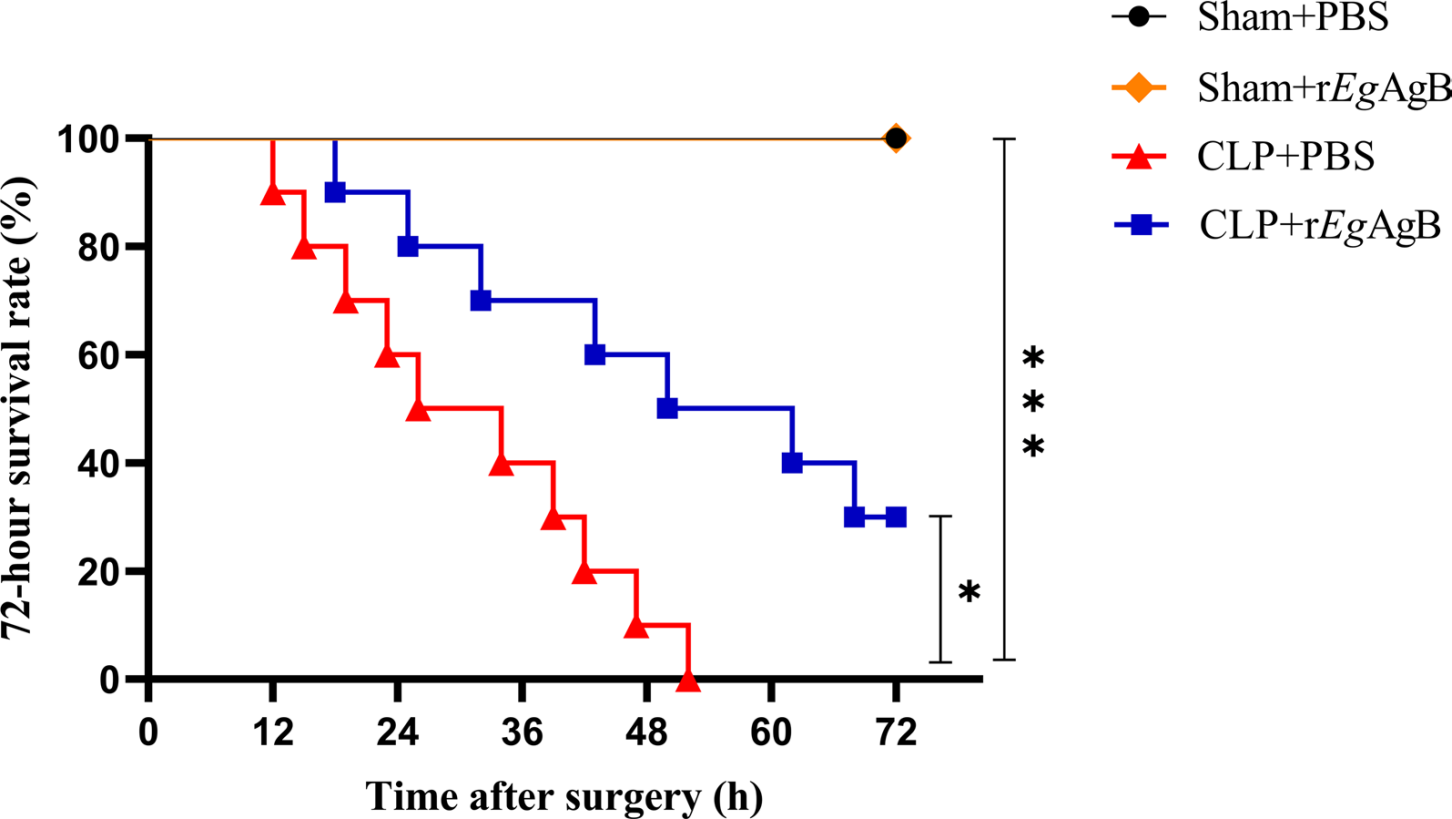
r*Eg*AgB treatment improved the survival rate of mice with CLP-induced sepsis. The survival rate was determined using Kaplan–Meier method and compared by log-rank test (*n* = 10 mice per group). *P < 0.05, **P < 0.01, ***P < 0.001

r*Eg*AgB was used to treat mice with CLP-induced sepsis to determine whether it improves the survival rate of septic mice. All mice in sham operation groups with or without r*Eg*AgB treatment (sham + r*Eg*AgB and sham + PBS) survived up for 72 h period, and all mice with CLP procedure without treatment died within 55 h after surgery (CLP + PBS). However, 40% of CLP surgery mice survived up to 72 h after being treated with 5 µg of r*Eg*AgB (CLP + r*Eg*AgB), which was significantly higher compared with the untreated CLP group (CLP + PBS) (Kaplan–Meier analysis: *χ*_2_ = 6.468, *df* = 1, *P* = 0.011). This result suggests that r*Eg*AgB treatment improves the survival rate of mice with sepsis (Fig. 3).

### Treatment with r*Eg*AgB inhibited proinflammatory cytokines and stimulated regulatory cytokines in mice with CLP-induced *sepsis*

**Fig. 4:**
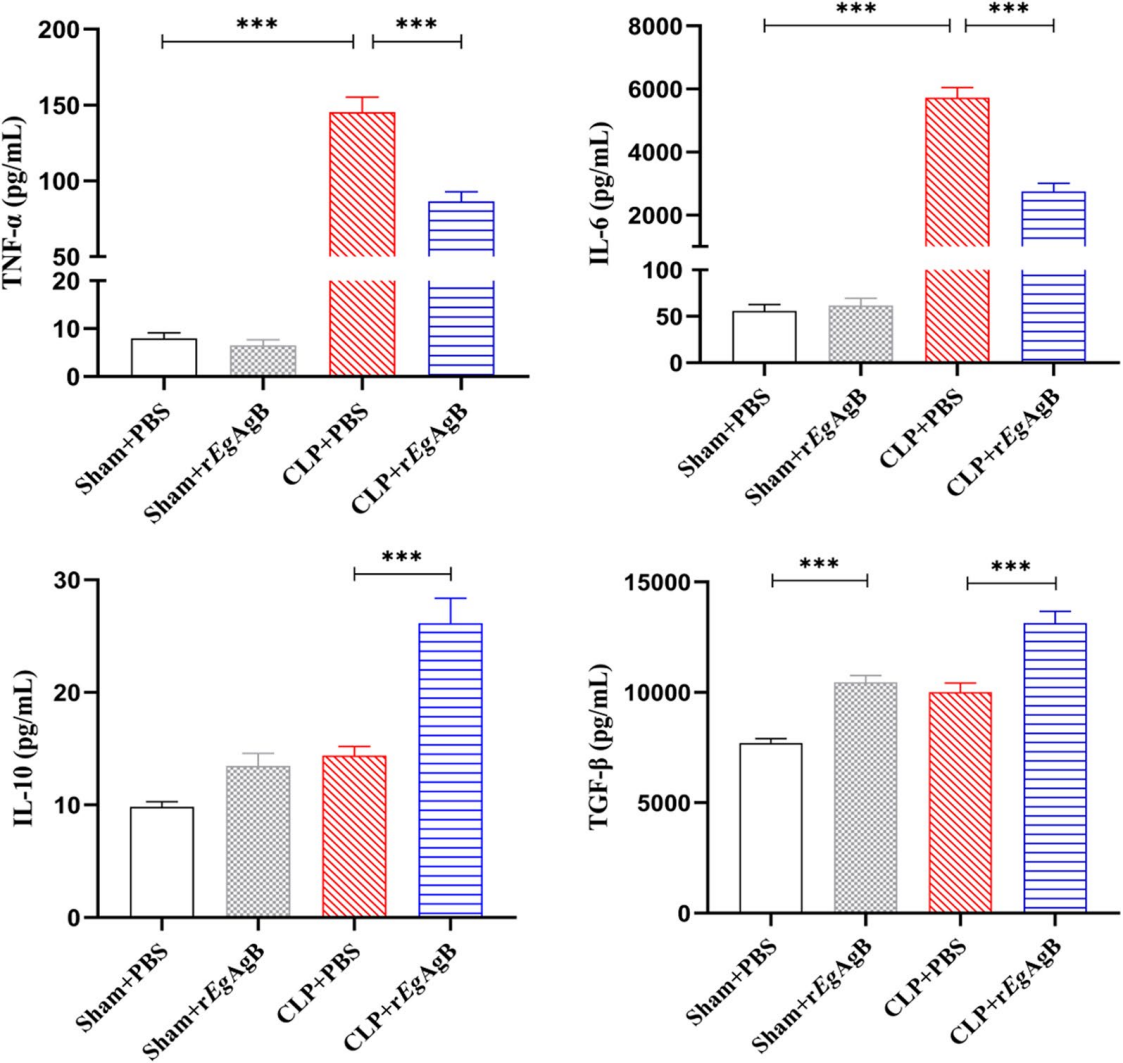
Treatment with r*Eg*AgB reduced the proinflammatory cytokines (TNF-α, IL-6) and induced the regulatory cytokines (IL-10, TGF-β) in sera of mice with CLP-induced sepsis measured by ELISA. The results were shown as the mean ± SEM for each group (*n* = 6). *P < 0.05, **P < 0.01, ***P < 0.001

CLP-induced sepsis causes inflammatory cytokine storm in mice that is the major cause of mortality [46]. To determine whether r*Eg*AgB improved survival rate of septic mice is related to the inhibition of the sepsis-induced inflammatory cytokine storm, the levels of proinflammatory cytokines (TNF-α, IL-6) and regulatory cytokines (IL-10, TGF-β) in the sera of each group of mice were measured. Compared with the mice in the sham + PBS control group, the serological levels of proinflammatory cytokine TNF-α and IL-6 were significantly increased in mice with CLP procedure (CLP + PBS group). After being treated with 5 µg of r*Eg*AgB, the levels of these proinflammatory cytokines in the sera of septic mice (CLP + r*Eg*AgB group) were significantly reduced compared with the septic group without treatment (CLP + PBS) (ANOVA: *F*_(3, 20)_ = 127.40, *P* < 0.0001; *F*_(3,20)_ = 169.20, *P* < 0.0001, respectively) (Fig. 4). Meanwhile, the serological levels of regulatory cytokines in the CLP + r*Eg*AgB treatment group were markedly higher than those in the group without r*Eg*AgB treatment (CLP + PBS) (ANOVA: *F*_(3, 20)_ = 39.28, *P* < 0.0001; *F*_(3,20)_ = 34.20, *P* < 0.0001, respectively). r*Eg*AgB itself had no effect on the production of proinflammatory or regulatory cytokines in mice with sham surgery (sham + r*Eg*AgB) (Fig. 4). These results suggest that r*Eg*AgB inhibits the release of proinflammatory cytokines and stimulates the secretion of regulatory cytokines.

### Treatment with r*Eg*AgB reduced the sepsis-caused pathological injury in liver, kidney, and lung of septic mice

**Fig. 5:**
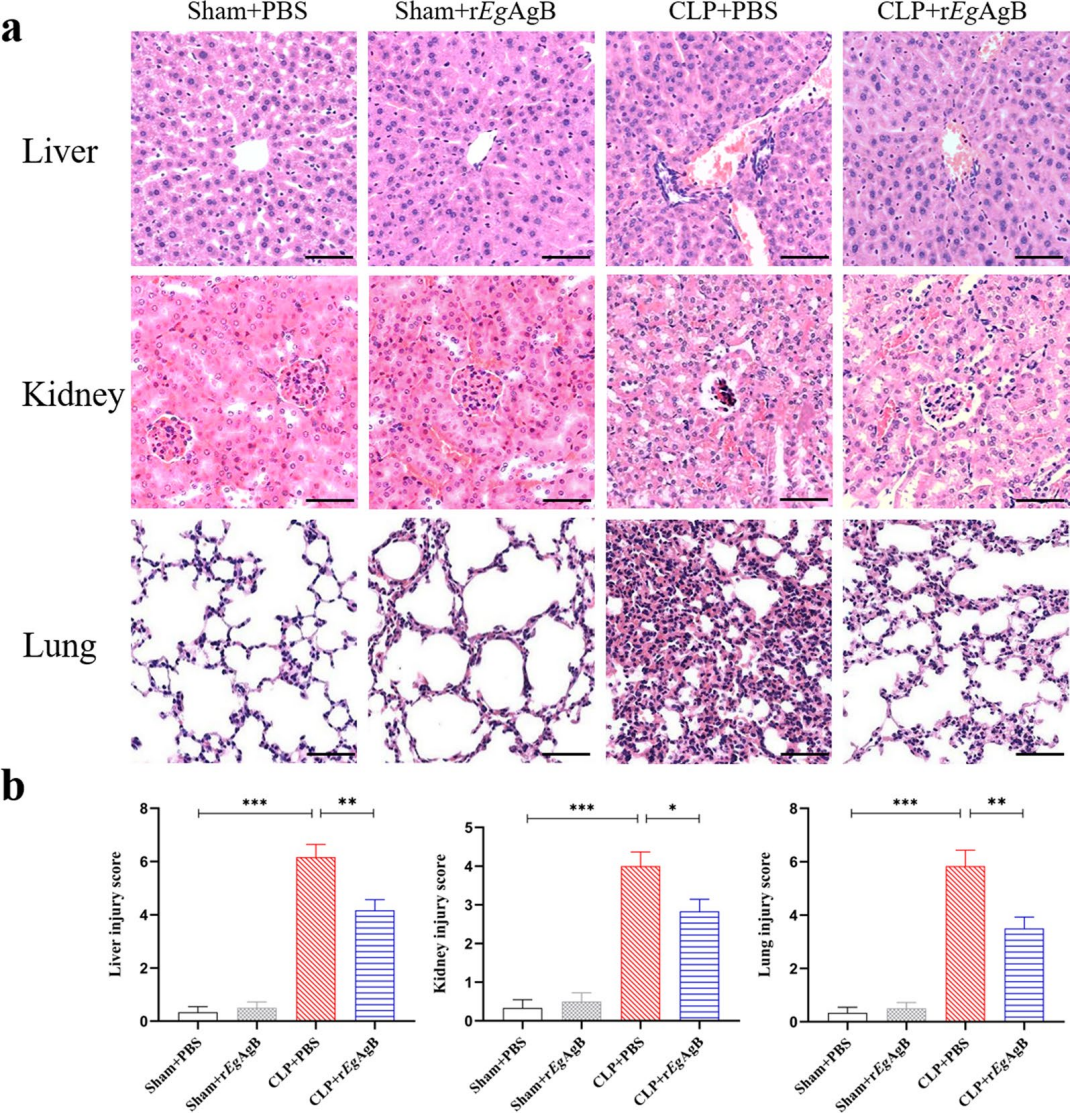
Treatment with r*Eg*AgB alleviated tissue injury in liver, kidney, and lung of mice with CLP-induced sepsis. **a** The histochemical sections of liver, kidney, and lung tissue in mice from different group. **b** Treatment with r*Eg*AgB improved injury score of liver, kidney, and lung in septic mice. The magnification × 200, scale bar = 100 µm. The results were shown as the mean ± SEM for each group (*n* = 6). *P < 0.05, **P < 0.01, ***P < 0.001

Sepsis causes tissue damage in vital organs of septic mice [29]. To determine the protective effect of r*Eg*AgB on the pathological damage caused by sepsis, the liver, kidney, and lung were collected from mice in each group and the histopathological changes in these tissues were observed under microscope. In mice with CLP-induced sepsis, the tissue of liver, kidney, and lung demonstrated significant injury including the disruption of tissue structure, infiltration of inflammatory cells, local necrosis, and congestion, or edema. Treatment with r*Eg*AgB significantly reduced the inflammation and tissue damage in septic mice (Fig. 5a). The pathological injury scores in liver, kidney, and lung of septic mice treated with r*Eg*AgB (CLP + r*Eg*AgB) were also remarkably reduced compared with mice without treatment (CLP + PBS) (ANOVA: *F*_(3, 20)_ = 77.09, *P* < 0.0001; *F*_(3,20)_ = 40.11, *P* < 0.0001;*F*_(3, 20)_ = 43.41, *P* < 0.0001, respectively) (Fig. 5b). There was not any pathological change in these organs from mice with sham procedure regardless of treatment (sham + PBS or sham + r*Eg*AgB).

**Fig. 6:**
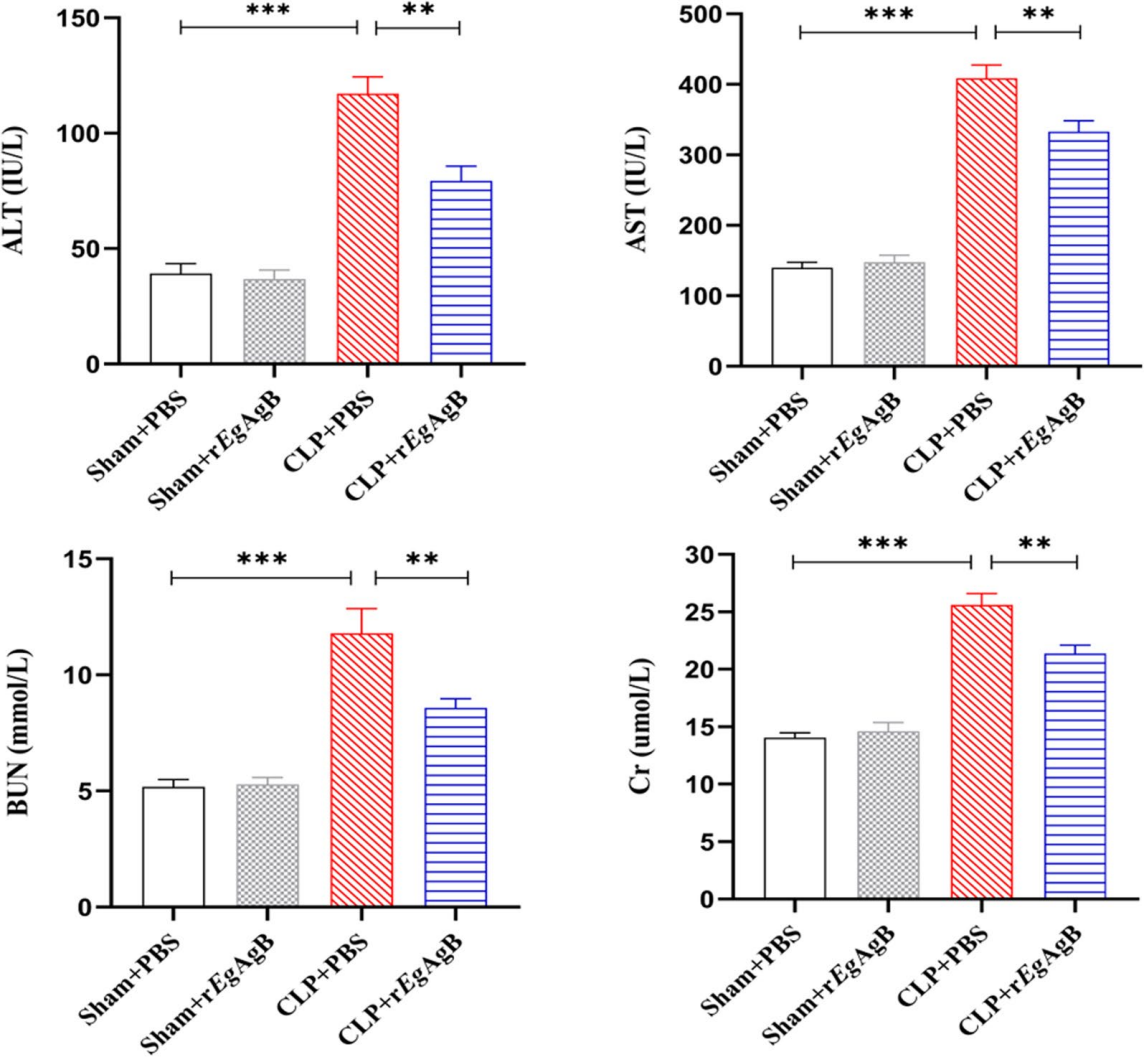
The serum levels of liver (ALT, AST) and kidney function markers (BUN, Cr) in mice of each group were measured by a fully automated biochemical analyzer. The results were shown as the mean ± SEM for each group (*n* = 6). *P < 0.05, **P < 0.01, ***P < 0.001

The serological levels of AST and ALT were used as markers for liver function damage, and the BUN and Cr measured for kidney function. Consistent with the tissue damage, the levels of these markers were significantly higher in the sera of mice with CLP operation compared with mice with sham operation, indicating that sepsis seriously damages the functions of liver and kidney (Fig. 6). However, treatment with r*Eg*AgB significantly decreased the levels of AST, ALT, BUN, and Cr in the sera of mice with CLP (CLP + r*Eg*AgB) compared with the group mice with CLP procedure without treatment (CLP + PBS) (ANOVA: *F*_(3, 16)_ = 97.97, *P* < 0.0001; *F*_(3, 16)_ = 45.89, *P* < 0.0001;*F*_(3, 16)_ = 26.95, *P* < 0.0001; *F*_(3, 16)_ = 54.41, *P* < 0.0001, respectively) (Fig. 6). The above results indicate that r*Eg*AgB is able to ameliorate key organ injury in septic mice and possess certain therapeutic or protective effect on CLP-induced sepsis in mice.

### Treatment with r*Eg*AgB reduced iNOS and boosted Arg-1 expression in liver, kidney, and lung tissues of septic mice

**Fig. 7:**
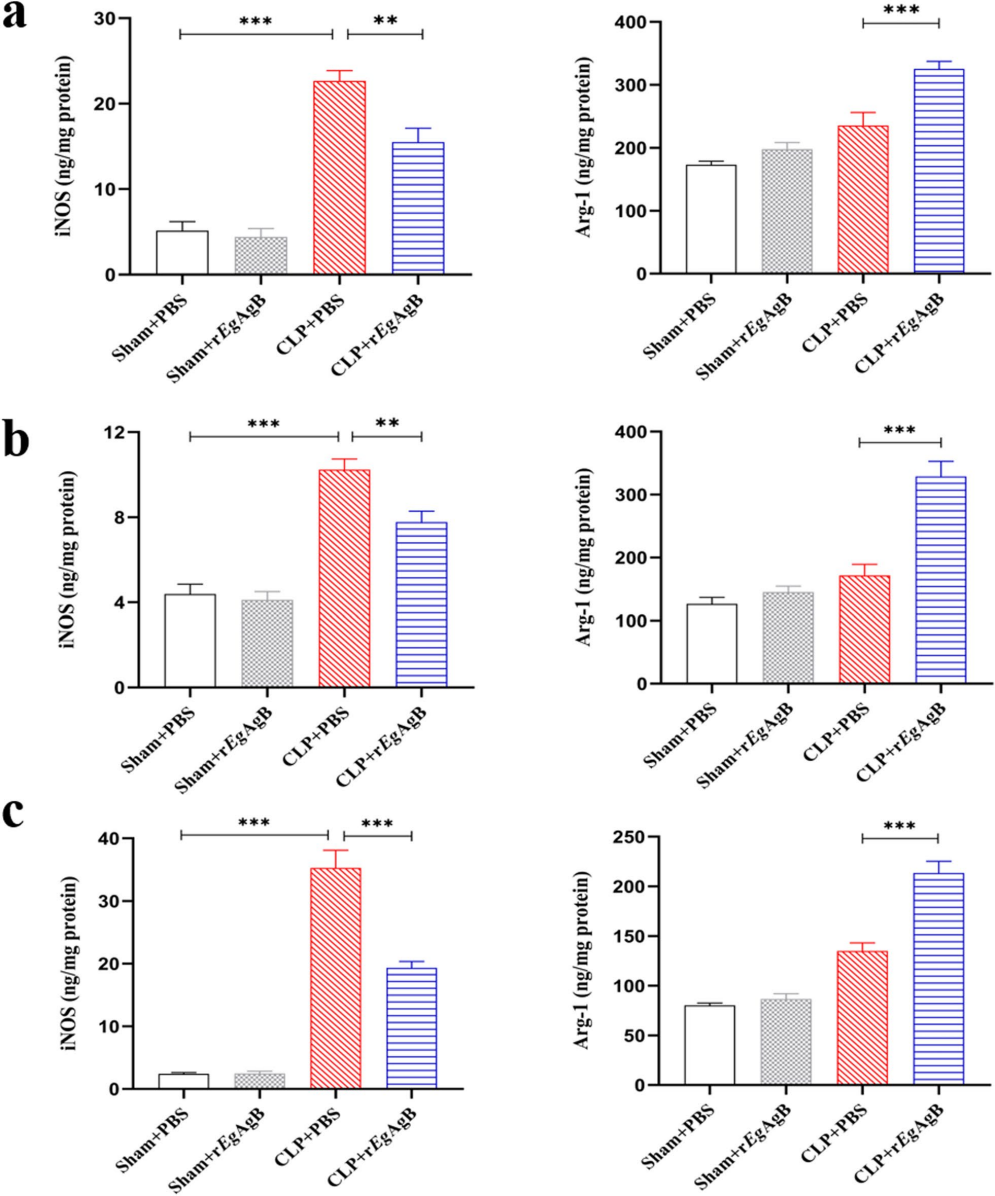
The expression levels of iNOS was reduced and Arg-1 increased in liver (**a**), kidney (**b**), and lung (**c**) tissues of septic mice treated with r*Eg*AgB. The results were shown as the mean ± SEM for each group (*n* = 6). *P < 0.05, **P < 0.01, ***P < 0.001

To further explore whether r*Eg*AgB ameliorates CLP-induced sepsis in mice by regulating inflammation in septic tissue, the protein expression levels of iNOS and Arg-1 were measured in liver, kidney, and lung tissue of septic mice using a ELISA kit. The results demonstrated that the expression level of iNOS in liver, kidney, and lung tissues of mice with CLP induced sepsis (CLP + PBS) was significantly increased compared with the mice with sham procedure (Fig. 7). The expression level of Arg-1 was also increased in mice with CLP but not as obvious as iNOS (Fig. 7). After being treated with r*Eg*AgB, the expression of iNOS was significantly reduced in these tissues of septic mice (CLP + r*Eg*AgB) compared with the group without treatment (CLP + PBS) (ANOVA: *F*_(3, 20)_ = 49.88, *P* < 0.0001; *F*_(3, 20)_ = 39.06, *P* < 0.0001;*F*_(3, 20)_ = 109.1, *P* < 0.0001, respectively) (Fig. 7). Noticeably, the expression of Arg-1 was significantly increased compared with group without treatment (CLP + PBS) (ANOVA: *F*_(3, 20)_ = 24.99, *P* < 0.0001; *F*_(3, 20)_ = 31.72, *P* < 0.0001;*F*_(3, 20)_ = 63.60, *P* < 0.0001, respectively) (Fig. 7). These results are consistent with the changes in serum inflammatory and regulatory cytokine levels, indicating that r*Eg*AgB alleviates sepsis by inhibiting the inflammatory response closely related to the polarization M1-like to M2-like.

### Incubation with r*Eg*AgB suppressed TLR2 and MyD88 on PMs

**Fig. 8:**
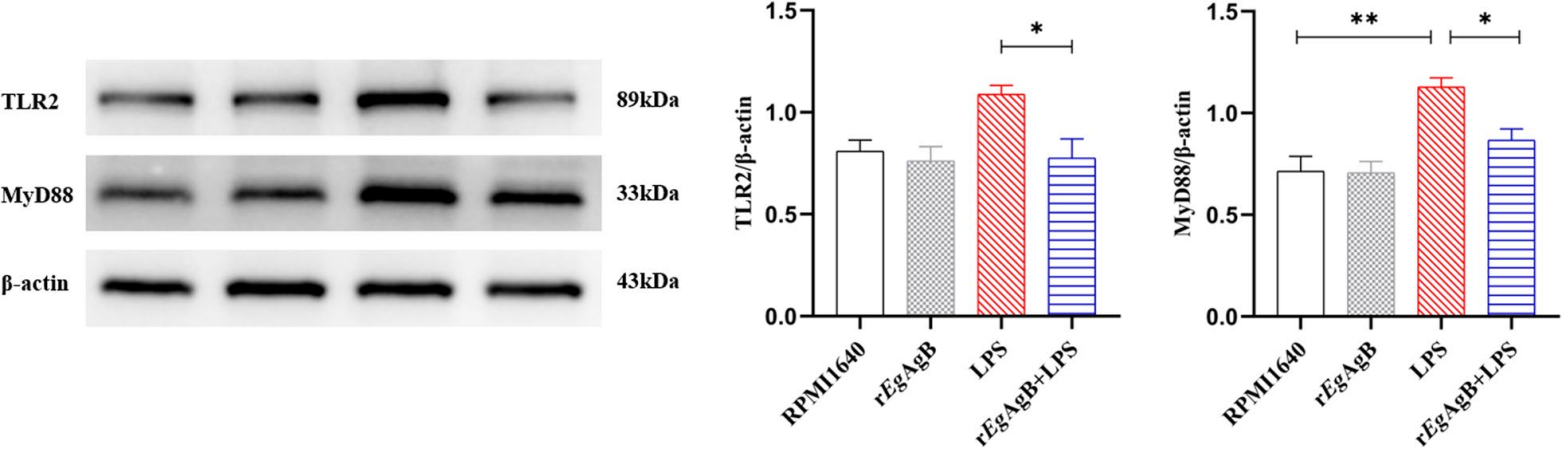
The expression of TLR2 and MyD88 in PMs detected by western blot. r*Eg*AgB downregulated TLR2 and MyD88 expression in LPS-induced PMs. β-actin was measured as a control. The results were shown as the mean ± SEM for each group (*n* = 3). *P < 0.05, **P < 0.01, ***P < 0.001

Recent studies suggested TLR2/MyD88 signal pathway played a critical role in modulation of macrophage polarization [47]. To determine whether TLR2/MyD88 signal pathway were involved in the r*Eg*AgB-triggered M2-like macrophage polarization, we evaluated the expression levels of TLR2 and MyD88 in PMs in each treatment group in vitro. The results demonstrated that LPS group contained higher protein expression levels of TLR2 and MyD88 compared with RPMI1640 group (Fig. 8). Co-incubation with r*Eg*AgB significantly decreased the protein expression levels of TLR2 and MyD88 in LPS + r*Eg*AgB group compared with LPS group (ANOVA: *F*_(3, 8)_ = 5.065, *P* = 0.0296; *F*_(3,8)_ = 11.70, *P* = 0.0027, respectively) (Fig. 8). These results indicated that r*Eg*AgB could downregulate TLR2/MyD88 signal pathway in PMs accompanied with reduced LPS-induced M1 polarization and increased M2-like polarization.

### Treatment with r*Eg*AgB alleviated polymicrobial sepsis associated with downregulation TLR2 and MyD88 pathway in septic mice

**Fig. 9:**
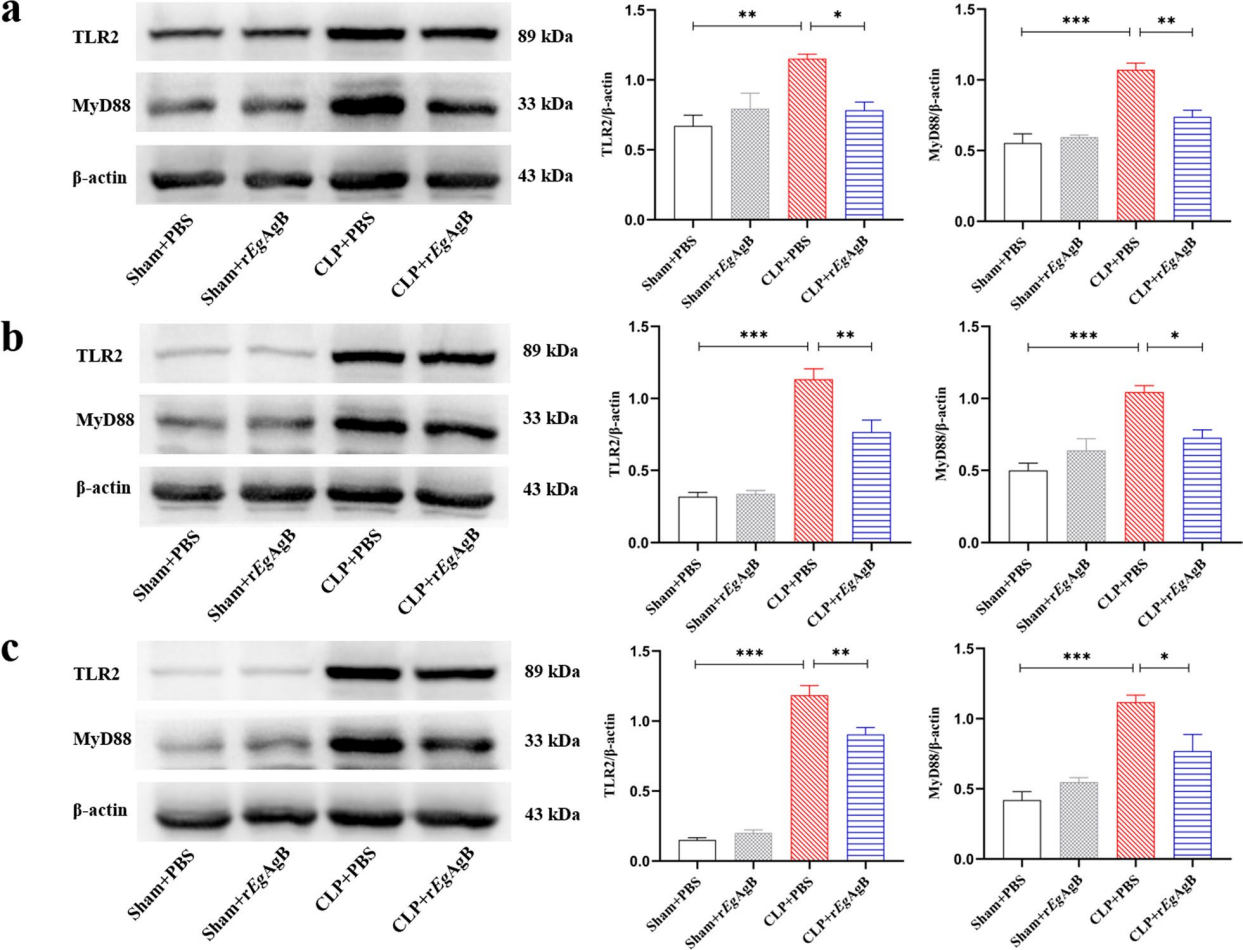
The expression of TLR2 and MyD88 in liver (**a**), kidney (**b**), and lung (**c**) of mice with CLP-induced sepsis detected by western blot. Treatment with r*Eg*AgB downregulated TLR2 and MyD88 expression in liver, kidney, and lung tissues of septic mice 12 h after CLP surgery. β-actin was measured as a control. The results were shown as the mean ± SEM for each group (*n* = 3). *P < 0.05, **P < 0.01, ***P < 0.001

We have identified that r*Eg*AgB could affect macrophage polarization through TLR2/MyD88 signal pathway in vitro (Figs. 1, 2, 8)*.* To investigate whether TLR2 and Myd88 signal pathway is involved in the treatment of r*Eg*AgB on sepsis, the expression levels of TLR2 and Myd88 in liver, kidney, and lung tissues were examined. The results showed that the expression of TLR2 and Myd88 in tissues of septic mice (CLP + PBS) was significantly increased compared with the mice with sham procedure (sham + PBS) (Fig. 9), while treatment with r*Eg*AgB effectively reduced the expression of TLR2 (ANOVA: *F*_(3, 8)_ = 7.784, *P* = 0.0093; *F*_(3,8)_ = 43.65, *P* < 0.0001; *F*_(3, 8)_ = 135.3, *P* < 0.0001, respectively) and Myd88 (ANOVA: *F*_(3, 8)_ = 24.71, *P* = 0.0002; *F*_(3,8)_ = 14.98, *P* = 0.0012; *F*_(3, 8)_ = 17.75, *P* = 0.0007, respectively) (Fig. 9), which was similar to the results found in PMs in vitro. The results indicate that treatment with r*Eg*AgB had the ability to regulate macrophage polarization possibly through suppressing TLR2 and MyD88 expressions, thus protecting tissues from being injured by the inflammatory storm of sepsis.

## Discussion

Sepsis is a systemic inflammatory response syndrome (SIRS) caused by serious bacterial infection. When infection occurs, pathogens multiply and release internal and external toxins such as LPS, which activate multiple inflammation-related signaling pathways and trigger inflammatory immune responses and the release of various inflammatory mediators [48]. Excessive inflammatory response and the imbalance of proinflammatory/antiinflammatory factors trigger systemic inflammatory response and cascade amplification, eventually leading to multiple key organ damage such as liver, kidney, and lung [49]. In this study, we successfully established a mouse sepsis model using the CLP procedure [50], and observed the damage to the structure and function of important organs such as liver, kidney, and lung, as well as the increased levels of proinflammatory cytokines in the blood of mice with CLP-induced sepsis. These results are consistent with the clinical manifestations of sepsis in clinical patients.

Our previous studies confirmed that *Eg*CF plays a role in promoting polarization of M2-type macrophages [38], and *Eg*AgB is the most immunogenic and abundant component of the protein in *Eg*CF [39]. It has been shown that *Eg*AgB is able to bind to the cell membranes of macrophages and monocytes, and induces a noninflammatory phenotype; in addition, *Eg*AgB has been shown to potentially treat inflammatory bowel disease by modulating macrophage differentiation towards M2-like [37, 39]. Thus we speculate that *Eg*AgB may affect macrophage polarization. It is known that *Eg*AgB is encoded by five gene subunits, of which *Eg*AgB subunit 2 is very immunogenic [51]. In this experiment we recombinantly expressed *Eg*AgB subunit 2 to observe its effect on macrophage polarization. Our preliminary results showed that incubation of r*Eg*AgB with PMs significantly inhibited LPS-induced M1-type polarization, meanwhile, IL-10 secretion was significantly increased in the culture supernatant, suggesting that M2-like macrophages were induced (Figs. 1, 2). As we know, macrophages are critically involved in the cleanup of invaded microbial infection and thereafter the pathology of sepsis [52, 53]. Since we have showed the regulation of r*Eg*AgB on macrophage M1/M2-like polarization in vitro, we would like to observe whether r*Eg*AgB is able to alleviate M1-dominated septic inflammation in vivo. Our results show that r*Eg*AgB treatment improves the survival rate of mice with sepsis (Fig. 3). Serological test also showed significantly reduced levels of the proinflammatory cytokines (TNF-α, IL-6) mainly secreted by M1 macrophages. On the contrary, the levels of IL-10 and TGF-β, the regulatory cytokines secreted mainly by the regulatory immune cells including M2 macrophages, were significantly increased (Fig. 4). The serological cytokine results are consistent with the in vitro results that show r*Eg*AgB stimulate M1/M2-like macrophage polarization. As we know, during microbial infection in sepsis, the first line of immune cells, including activated M1 macrophages, secreted proinflammatory cytokines that further amplify the inflammatory responses, leading to the multiple organ damage while controlling the infection. M2 macrophages secrete regulatory or antiinflammatory cytokines such as IL-10 and TGF-β to reduce excessive inflammation and promote tissue repair [54, 55]. The histopathological examination on the key organs, including livers, kidneys, and lungs, of septic mice demonstrated significantly reduced infiltration of inflammatory cells and tissue damage after being treated with r*Eg*AgB (Fig. 5). The biomarkers of liver damage (ALT and AST) and kidney function (BUN and Cr) were also significantly improved (Fig. 6). These improved pathological results may explain the increased survival rate in r*Eg*AgB-treated septic mice. The improved immunopathological changes are also correlated with the decrease M1 macrophages marker iNOS and increased M2 macrophages marker Arg-1 in these tissues of mice after being treated with r*Eg*AgB (Fig. 7), further indicating that the therapeutic effect of r*Eg*AgB on the inflammatory sepsis is associated with the M1/M2-like polarization systematically or locally in these impacted key organs. However, we cannot exclude the possibility of the expression of Arg-1 and iNOS by other cells in tissues besides macrophages. Therefore, test on the macrophages isolated from these tissues would provide direct evidence of effect of r*Eg*AgB on the local macrophages, and it has been included in our next experiment. The above results suggest that M1 macrophages play an important role in the pathogenesis of sepsis, therefore, inhibiting the activation of M1 macrophages and inducing M2-like macrophages may be involved in the reduced excessive activation of inflammatory responses in sepsis.

There is increasing evidence that demonstrates that helminth infection or helminth-derived proteins can ameliorate hyperinflammation by inhibiting Th1/Th17 responses and inducing Th2-type response, including the phenotypic changes of macrophage from M1 to M2 [56–58]. The results in this study are consistent with our previous studies that showed *Schistosoma japonicum*-secreted cysteine protease inhibitor (r*Sj*-Cys) and secretory/excretory products from *Trichinella spiralis* adult worms (*Ts*-AES) ameliorated sepsis-caused tissue pathology and damage through similar immunomodulatory mechanism targeting M1/M2 macrophage polarization [28, 59]. The immunomodulatory property of *E. granulosus* infection and its cyst fluid proteins on human immune system have been demonstrated previously in the treatment of ovalbumin (OVA)-induced asthma in mice by increasing IL-10 and Tregs and down-regulating IL-5, IL-17 [60–62]. As the main antigen secreted by *E. granulosus*, *Eg*AgB exhibits a series of immunomodulatory properties and regulates a variety of innate immune system cells, including lymphocytes, neutrophils, monocytes, and dendritic cells [35, 63–65]. The results in this study further confirm that *E. coli*-expressed soluble recombinant *Eg*AgB protein exhibits similar immunomodulatory effects of the native *E. granulosus* on the M1/M2 polarization and reduces inflammation activated by serious bacterial infection.

During bacterial infection, TLRs on the macrophage and other immune cells play important roles in the recognition of LPS on bacteria and initiating the immune responses to clean the invaded microbial pathogens [24, 65], in which TLR2 recognizes a wide range of microbial ligands and is one of the powerful targets for the treatment of sepsis [66]. The TLR-mediated cascade immune response use Myd88 as a bridge to transmit inflammatory signals downstream, which leads to the activation of nuclear transcription factor NF-κB and further regulates the expression of inflammatory genes such as TNF-α and IL-6 [67]. Previous studies have shown that helminth-derived proteins regulate macrophages through inhibiting TLR2/MyD88 pathway [46, 59]. In this study, we demonstrated that the reduced inflammation and tissue injury in septic group treated with r*Eg*AgB was consistent with reduced expression levels of TLR2 and MyD88 in liver, kidney, and lung tissues, indicating that immunomodulation of r*Eg*AgB may take effect through inhibition of TLR2/MyD88 pathway. Combined with the results of in vitro studies on macrophages, the potential mechanism by which r*Eg*AgB ameliorates sepsis-induced hyperinflammatory response may be to shift the M1-like to M2-like phenotype of macrophages through inhibiting TLR2 and MyD88 pathways. However, the detail inflammatory pathway affected by *Eg*AgB including TLRs/MyD88/NF-κB should be further investigated.

## Conclusions

In this study, we demonstrated the important roles of inflammatory macrophages in the pathogenesis of sepsis. r*Eg*AgB subunit 2 was able to inhibit the excessive inflammatory response, thereby attenuating the immunopathological damage of vital organs such as liver, kidney, and lung in septic mice. This effect may go through immunomodulating host immune responses by inhibiting the production of proinflammatory cytokines and inducing regulatory cytokines. The mechanism underlying the immunomodulatory effect of r*Eg*AgB subunit 2 possibly involves the polarization of macrophages from proinflammatory M1 to regulatory M2 phenotype through inhibiting TLR2/MyD88 inflammatory pathway. This provides a clear direction for our future research and also suggests that *Eg*AgB subunit 2 may play an important role in the modulation and regulation of host immune responses as an immunological escape strategy.

## Supplementary Information

Supplementary material 1: Figure 1: SDS-PAGE of purified rEgAgB. Total 3 μg of purified rEgAgB was separated by 12% polyacrylamide gel electrophoresis

## Data Availability

No datasets were generated or analysed during the current study.

## Abbreviations

SIRS: Systemic inflammatory response syndrome
*Eg*AgB: *Echinococcus granulosus* Hydatid cyst-secreted antigen B
CLP: Cecal ligation and puncture
AAM: Alternatively activated macrophages
TLRs: Toll-like receptors
PAMP: Pathogen-associated molecular patterns
Tregs: Regulatory T cells
CE: Cystic echinococcosis
HLBP: Hydrophobic ligand binding protein
*Eg*CF: *E. granulosus* Cyst fuid
IMAC: Immobilized metal ion affinity chromatography
PMs: Peritoneal macrophages
ALT: Alanine transaminase
AST: Aspartate transaminase
BUN: Blood urea nitrogen
Cr: Creatinine
HE: Hematoxylin and eosin
PVDF: Polyvinylidene fluoride

## References

[1] Mayr F, Yende S, Angus D. Epidemiology of severe sepsis. Virulence. 2014;5:4–11. 24335434 10.4161/viru.27372 PMC3916382

[2] Singer M, Deutschman CS, Seymour CW, Shankar-Hari M, Annane D, Bauer M, et al. The thirdinternational consensus definitions for sepsis and septic shock (Sepsis-3). JAMA. 2016;315:801–10. 26903338 10.1001/jama.2016.0287 PMC4968574

[3] Rudd KE, Johnson SC, Agesa KM, Shackelford KA, Tsoi D, Kievlan DR, et al. Global, regional, and national sepsis incidence and mortality, 1990–2017: analysis for the Global Burden of Disease Study. Lancet. 2020;395:200–11. 31954465 10.1016/S0140-6736(19)32989-7 PMC6970225

[4] Fleischmann C, Scherag A, Adhikari NK, Hartog CS, Tsaganos T, Schlattmann P, et al. Assessment of global incidence and mortality of hospital-treated sepsis. Current estimates and limitations. Am J Respir Crit Care Med. 2016;193:259–72. 26414292 10.1164/rccm.201504-0781OC

[5] Savio LEB, de Andrade MP, Figliuolo VR, de Avelar Almeida TF, Santana PT, Oliveira SDS, et al. CD39 limits P2X7 receptor inflammatory signaling and attenuates sepsis-induced liver injury. J Hepatol. 2017;67:716–26. 28554875 10.1016/j.jhep.2017.05.021 PMC5875702

[6] Deng Z, He M, Hu H, Zhang W, Zhang Y, Ge Y et al. Melatonin attenuates sepsis-induced acute kidney injury by promoting mitophagy through SIRT3-mediated TFAM deacetylation. Autophagy. 2024;20:151–65. 37651673 10.1080/15548627.2023.2252265 PMC10761103

[7] Drechsler S, Weixelbaumer KM, Weidinger A, Raeven P, Khadem A, Redl H, et al. Why do they die? Comparison of selected aspects of organ injury and dysfunction in mice surviving and dying in acute abdominal sepsis. Intensive Care Med Exp. 2015;3:48. 26215812 10.1186/s40635-015-0048-z PMC4513036

[8] Huang M, Cai S, Su J. The pathogenesis of sepsis and potential therapeutic targets. Int J Mol Sci. 2019;20:5376. 31671729 10.3390/ijms20215376 PMC6862039

[9] Karakike E, Giamarellos-Bourboulis EJ. Macrophage activation-like syndrome: a distinct entity leading to early death in sepsis. Front Immunol. 2019;10:55. 30766533 10.3389/fimmu.2019.00055 PMC6365431

[10] Li Y, Zhang H, Chen C, Qiao K, Li Z, Han J, et al. Biomimetic immunosuppressive exosomes that inhibit cytokine storms contribute to the alleviation of sepsis. Adv Mater. 2022;34:e2108476. 35267211 10.1002/adma.202108476

[11] Almalki WH. The sepsis induced defective aggravation of immune cells: a translational science underling chemico-biological interactions from altered bioenergetics and/or cellular metabolism to organ dysfunction. Mol Cell Biochem. 2021;476:2337–44. 33586093 10.1007/s11010-021-04066-9

[12] Ardura JA, Rackov G, Izquierdo E, Alonso V, Gortazar AR, Escribese MM. Targeting macrophages: friends or foes in disease? Front Pharmacol. 2019;10:1255. 31708781 10.3389/fphar.2019.01255 PMC6819424

[13] Abdelaziz MH, Abdelwahab SF, Wan J, Cai W, Huixuan W, Jianjun C, et al. Alternatively activated macrophages; a double-edged sword in allergic asthma. J Transl Med. 2020;18:58. 32024540 10.1186/s12967-020-02251-w PMC7003359

[14] Liu YC, Zou XB, Chai YF, Yao YM. Macrophage polarization in inflammatory diseases. Int J Biol Sci. 2014;10:520–9. 24910531 10.7150/ijbs.8879 PMC4046879

[15] Kumar V. Targeting macrophage immunometabolism: dawn in the darkness of sepsis. Int Immunopharmacol. 2018;58:173–85. 29625385 10.1016/j.intimp.2018.03.005

[16] Liang X, Li T, Zhou Q, Pi S, Li Y, Chen X, et al. Mesenchymal stem cells attenuate sepsis-induced liver injury via inhibiting M1 polarization of Kupffer cells. Mol Cell Biochem. 2019;452:187–97. 30178273 10.1007/s11010-018-3424-7

[17] Mantovani A, Sozzani S, Locati M, Allavena P, Sica A. Macrophage polarization: tumor-associated macrophages as a paradigm for polarized M2 mononuclear phagocytes. Trends Immunol. 2002;23:549–55. 12401408 10.1016/s1471-4906(02)02302-5

[18] Martinez FO, Gordon S. The M1 and M2 paradigm of macrophage activation: time for reassessment. F1000prime Rep. 2014;6:13. 24669294 10.12703/P6-13 PMC3944738

[19] Wang S, Wang J, Chen Z, Luo J, Guo W, Sun L, et al. Targeting M2-like tumor-associated macrophages is a potential therapeutic approach to overcome antitumor drug resistance. NPJ Precis Oncol. 2024;8:31. 38341519 10.1038/s41698-024-00522-z PMC10858952

[20] Wynn TA, Chawla A, Pollard JW. Macrophage biology in development, homeostasis and disease. Nature. 2013;496:445–55. 23619691 10.1038/nature12034 PMC3725458

[21] Dang CP, Leelahavanichkul A. Over-expression of miR-223 induces M2 macrophage through glycolysis alteration and attenuates LPS-induced sepsis mouse model, the cell-based therapy in sepsis. PLoS ONE. 2020;15:e0236038. 32658933 10.1371/journal.pone.0236038 PMC7357756

[22] Yao M, Cui B, Zhang W, Ma W, Zhao G, Xing L. Exosomal miR-21 secreted by IL-1β-primed-mesenchymal stem cells induces macrophage M2 polarization and ameliorates sepsis. Life Sci. 2021;264:118658. 33115604 10.1016/j.lfs.2020.118658

[23] Jiménez-Dalmaroni MJ, Gerswhin ME, Adamopoulos IE. The critical role of toll-like receptors–From microbial recognition to autoimmunity: a comprehensive review. Autoimmun Rev. 2016;15:1–8. 26299984 10.1016/j.autrev.2015.08.009 PMC4679489

[24] Kumar V. Toll-like receptors in sepsis-associated cytokine storm and their endogenous negative regulators as future immunomodulatory targets. Int Immunopharmacol. 2020;89:107087. 33075714 10.1016/j.intimp.2020.107087 PMC7550173

[25] Yang J, Liu W, Xu M, Yu L. Long non-coding RNA CRNDE and toll-like receptor 3 correlate with disease severity, inflammation, and mortality in sepsis. J Clin Lab Anal. 2020;34:e23360. 32696505 10.1002/jcla.23360 PMC7521289

[26] Zhang X, Zhang M, Zhou M, Zhang T, Gao Y, Li S, et al. Tetrahedral-framework nucleic acids carry small interfering rna to downregulate toll-like receptor 2 gene expression for the treatment of sepsis. ACS Appl Mater Interfaces. 2022;14:6442–52. 35080860 10.1021/acsami.1c23708

[27] Wang M, Wu L, Weng R, Zheng W, Wu Z, Lv Z. Therapeutic potential of helminths in autoimmune diseases: helminth-derived immune-regulators and immune balance. Parasitol Res. 2017;116:2065–74. 28664463 10.1007/s00436-017-5544-5

[28] Gao X, Mao C, Zheng T, Xu X, Luo X, Zhang S, et al.* Schistosoma japonicum*-derived peptide SJMHE1 ameliorates allergic symptoms and responses in mice with allergic rhinitis. Front Cell Infect Microbiol. 2023;13:1143950. 37346033 10.3389/fcimb.2023.1143950 PMC10279851

[29] Xie H, Wu L, Chen X, Gao S, Li H, Yuan Y, et al. *Schistosoma japonicum* cystatin alleviates sepsis through activating regulatory macrophages. Front Cell Infect Microbiol. 2021;11:617461. 33718268 10.3389/fcimb.2021.617461 PMC7943722

[30] Yang H, Li H, Chen W, Mei Z, Yuan Y, Wang X, et al. Therapeutic effect of *Schistosoma japonicum* cystatin on atherosclerotic renal damage. Front Cell Dev Biol. 2021;9:760980. 34901005 10.3389/fcell.2021.760980 PMC8656285

[31] Okakpu OK, Dillman AR. Review of the role of parasitic nematode excretory/secretory proteins in host immunomodulation. J Parasitol. 2022;108:199–208. 35435987 10.1645/21-33

[32] Hübner MP, Layland LE, Hoerauf A. Helminths and their implication in sepsis - A new branch of their immunomodulatory behaviour. Pathog Dis. 2013;69:127–41. 23929557 10.1111/2049-632X.12080 PMC4285315

[33] Woolsey ID, Miller AL. *Echinococcus granulosus* sensu lato and *Echinococcus multilocularis*: a review. Res Vet Sci. 2021;135:517–22. 33246571 10.1016/j.rvsc.2020.11.010

[34] Wang Y, Ma BC, Wang LY, Quzhen G, Pang HS. Effects of management of infection source of echinococcosis in Linzhi, Tibet Autonomous Region of China. Infect Dis Poverty. 2021;10:25. 33676564 10.1186/s40249-021-00805-8 PMC7936428

[35] Mamuti W, Sako Y, Nakao M, Xiao N, Nakaya K, Ishikawa Y, et al. Recent advances in characterization of *Echinococcus* antigen B. Parasitol Int. 2006;55:S57-62. 16360336 10.1016/j.parint.2005.11.008

[36] Rialch A, Raina OK, Tigga MN, Anandanarayanan A, Ganaie ZA, Aftab A, et al. Evaluation of *Echinococcus granulosus* recombinant EgAgB8/1, EgAgB8/2 and EPC1 antigens in the diagnosis of cystic echinococcosis in buffaloes. Vet Parasitol. 2018;252:29–34. 29559147 10.1016/j.vetpar.2018.01.020

[37] Silva-Álvarez V, Folle AM, Ramos AL, Kitano ES, Iwai LK, Corraliza I, et al. *Echinococcus granulosus* Antigen B binds to monocytes and macrophages modulating cell response to inflammation. Parasit Vectors. 2016;9:69. 26846700 10.1186/s13071-016-1350-7 PMC4743400

[38] Wang S, Jiang D, Huang F, Qian Y, Qi M, Li H, et al. Therapeutic effect of *Echinococcus granulosus* cyst fluid on bacterial sepsis in mice. Parasit Vectors. 2023;16:450. 38066526 10.1186/s13071-023-06021-7 PMC10709918

[39] Bao J, Qi W, Sun C, Tian M, Jiao H, Guo G, et al.*Echinococcus granulosus*sensu stricto and antigen B may decrease inflammatory bowel disease through regulation of M1/2 polarization. Parasit Vectors. 2022;15:391. 36289514 10.1186/s13071-022-05498-y PMC9608937

[40] Zhang W, Li J, Jones MK, Zhang Z, Zhao L, Blair D, et al. The *Echinococcus granulosus* antigen B gene family comprises at least 10 unique genes in five subclasses which are differentially expressed. PLoSNegl Trop Dis. 2010;4:e784. 10.1371/journal.pntd.0000784 PMC2919375 20706625

[41] Cassado Ados A, D’Império Lima MR, Bortoluci KR. Revisiting mouse peritoneal macrophages: heterogeneity, development, and function. Front Immunol. 2015;6:225. 26042120 10.3389/fimmu.2015.00225 PMC4437037

[42] Li H, Wang S, Zhan B, He W, Chu L, Qiu D, et al. Therapeutic effect of *Schistosoma japonicum* cystatin on bacterial sepsis in mice. Parasit Vectors. 2017;10:222. 28482922 10.1186/s13071-017-2162-0 PMC5422996

[43] Dejager L, Pinheiro I, Dejonckheere E, Libert C. Cecal ligation and puncture: the gold standard model for polymicrobial sepsis? Trends Microbiol. 2011;19:198–208. 21296575 10.1016/j.tim.2011.01.001

[44] Li YF, Xu BY, An R, Du XF, Yu K, Sun JH, et al. Protective effect of anisodamine in rats with glycerol-induced acute kidney injury. BMC Nephrol. 2019;20:223. 31208365 10.1186/s12882-019-1394-y PMC6580578

[45] Wang L, Cao Y, Gorshkov B, Zhou Y, Yang Q, Xu J, et al. Ablation of endothelial Pfkfb3 protects mice from acute lung injury in LPS-induced endotoxemia. Pharmacol Res. 2019;146:104292. 31167111 10.1016/j.phrs.2019.104292 PMC7310404

[46] Li H, Qiu D, Yuan Y, Wang X, Wu F, Yang H, et al. *Trichinella spiralis* cystatin alleviates polymicrobial sepsis through activating regulatory macrophages. Int Immunopharmacol. 2022;109:108907. 35691271 10.1016/j.intimp.2022.108907

[47] Wang B, Wu Y, Liu R, Xu H, Mei X, Shang Q, et al. Lactobacillus rhamnosus GG promotes M1 polarization in murine bone marrow-derived macrophages by activating TLR2/MyD88/MAPK signaling pathway. Anim Sci J. 2020;91:e13439. 32779289 10.1111/asj.13439

[48] Lelubre C, Vincent JL. Mechanisms and treatment of organ failure in sepsis. Nat Rev Nephrol. 2018;14:417–27. 29691495 10.1038/s41581-018-0005-7

[49] Ni J, Zhao Y, Su J, Liu Z, Fang S, Li L, et al. Toddalolactone protects lipopolysaccharide-induced sepsis and attenuates lipopolysaccharide-induced inflammatory response by modulating HMGB1-NF-κB translocation. Front Pharmacol. 2020;11:109. 32153412 10.3389/fphar.2020.00109 PMC7047824

[50] Drechsler S, Osuchowski M. Cecal ligation and puncture. Methods Mol Biol. 2021;2321:1–8. 34048002 10.1007/978-1-0716-1488-4_1

[51] Bashiri S, Nemati Mansoor F, Valadkhani Z. Expansion of a highly sensitive and specific ELISA test for diagnosis of hydatidosis using recombinant *Eg*B8/2 protein. Iran J Basic Med Sci. 2019;22:134–9. 30834077 10.22038/ijbms.2018.29024.7021 PMC6396996

[52] Chen C, Zhao D, Fang S, Chen Q, Cheng B, Fang X, et al. TRIM22-mediated apoptosis is associated with Bak oligomerization in monocytes. Sci Rep. 2017;7:39961. 28079123 10.1038/srep39961 PMC5228056

[53] Xie DL, Zheng MM, Zheng Y, Gao H, Zhang J, Zhang T, et al. Vibrio vulnificus induces mTOR activation and inflammatory responses in macrophages. PLoS ONE. 2017;12:e0181454. 28719654 10.1371/journal.pone.0181454 PMC5515453

[54] Funes SC, Rios M, Escobar-Vera J, Kalergis AM. Implications of macrophage polarization in autoimmunity. Immunology. 2018;154:186–95. 29455468 10.1111/imm.12910 PMC5980179

[55] Shapouri-Moghaddam A, Mohammadian S, Vazini H, Taghadosi M, Esmaeili SA, Mardani F, et al. Macrophage plasticity, polarization, and function in health and disease. J Cell Physiol. 2018;233:6425–40. 29319160 10.1002/jcp.26429 PMC13482560

[56] Schramm G, Suwandi A, Galeev A, Sharma S, Braun J, Claes AK, et al. *Schistosome* eggs impair protective Th1/Th17 immune responses against *salmonella* infection. Front Immunol. 2018;9:2614. 30487793 10.3389/fimmu.2018.02614 PMC6246638

[57] Radovic I, Gruden-Movsesijan A, Ilic N, Cvetkovic J, Mojsilovic S, Devic M, et al. Immunomodulatory effects of *Trichinella spirali*s-derived excretory-secretory antigens. Immunol Res. 2015;61:312–25. 25616617 10.1007/s12026-015-8626-4

[58] Ho CH, Cheng CH, Huang TW, Peng SY, Lee KM, Cheng PC. Switched phenotypes of macrophages during the different stages of *Schistosoma japonicum* infection influenced the subsequent trends of immune responses. J Microbiol Immunol Infect. 2022;55:503–26. 34330662 10.1016/j.jmii.2021.06.005

[59] Li H, Qiu D, Yang H, Yuan Y, Wu L, Chu L, et al. Therapeutic efficacy of excretory-secretory products of *Trichinella spiralis* adult worms on sepsis-induced acute lung injury in a mouse model. Front Cell Infect Microbiol. 2021;11:653843. 33842398 10.3389/fcimb.2021.653843 PMC8024484

[60] Wang H, Li J, Pu H, Hasan B, Ma J, Jones MK, et al. *Echinococcus granulosus* infection reduces airway inflammation of mice likely through enhancing IL-10 and down-regulation of IL-5 and IL-17A. Parasit Vectors. 2014;7:522. 25409540 10.1186/s13071-014-0522-6 PMC4256745

[61] Kim HJ, Kang SA, Yong TS, Shin MH, Lee KJ, Park GM, et al. Therapeutic effects of *Echinococcus granulosus* cystic fluid on allergic airway inflammation. Exp Parasitol. 2019;198:63–70. 30763570 10.1016/j.exppara.2019.02.003

[62] Jeong MJ, Kang SA, Choi JH, Lee DI, Yu HS. Extracellular vesicles of *Echinococcus granulosus* have therapeutic effects in allergic airway inflammation. Parasite Immunol. 2021;43:e12872. 34174101 10.1111/pim.12872

[63] Siracusano A, Margutti P, Delunardo F, Profumo E, Riganò R, Buttari B, et al. Molecular cross-talk in host-parasite relationships: the intriguing immunomodulatory role of *Echinococcus* antigen B in cystic echinococcosis. Int J Parasitol. 2008;38:1371–6. 18692060 10.1016/j.ijpara.2008.06.003

[64] Riganò R, Buttari B, Profumo E, Ortona E, Delunardo F, Margutti P, et al. *Echinococcus granulosus* antigen B impairs human dendritic cell differentiation and polarizes immature dendritic cell maturation towards a Th2 cell response. Infect Immun. 2007;75:1667–78. 17210662 10.1128/IAI.01156-06 PMC1865679

[65] Ludwig-Portugall I, Layland LE. TLRs, Treg, and B Cells, an interplay of regulation during helminth infection. Front Immunol. 2012;3:8. 22566894 10.3389/fimmu.2012.00008 PMC3342019

[66] Sato M, Takeuchi S, Moriya R, Kito T, Soga S, Aoyama K, et al. Novel TLR2xTLR4 bispecific antibody inhibits bacterial sepsis. Monoclon Antib Immunodiagn Immunother. 2021;40:6–10. 33347385 10.1089/mab.2020.0025

[67] Castoldi A, Braga TT, Correa-Costa M, Aguiar CF, Bassi ÊJ, Correa-Silva R, et al. TLR2, TLR4 and the MYD88 signaling pathway are crucial for neutrophil migration in acute kidney injury induced by sepsis. PLoS ONE. 2012;7:e37584. 22655058 10.1371/journal.pone.0037584 PMC3360043

