# Supplementary figures and images for: Therapeutic effect of recombinant Echinococcus granulosus antigen B subunit 2 protein on sepsis in a mouse model
Source: Parasit Vectors. 2024 Nov 15;17:467. doi: 10.1186/s13071-024-06540-x (PMC11566433; doi:10.1186/s13071-024-06540-x)

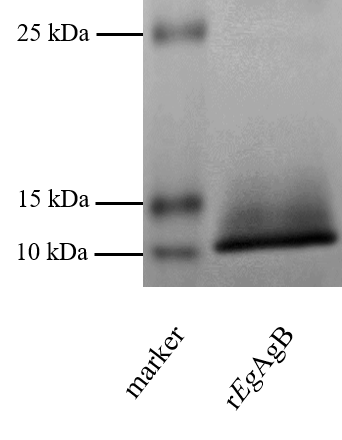

Supplement: Supplementary file 1 — Supplementary material 1: Figure 1: SDS-PAGE of purified rEgAgB. Total 3 μg of purified rEgAgB was separated by 12% polyacrylamide gel electrophoresis [file 13071_2024_6540_MOESM1_ESM.tif]
